# Use of Different Process Gases for Manufacturing Isolating Alumina Coatings by Flame Spraying with Cords

**DOI:** 10.1007/s11666-021-01160-8

**Published:** 2021-02-05

**Authors:** Michél Hauer, Melanie Meyer, Dominique Billieres, Cédric Bricquet, Franz Gerstgrasser, Jarkko Kiilakoski, Julien Lejay, Knuth-Michael Henkel

**Affiliations:** 1 Fraunhofer Institute for Large Structures in Production Engineering IGP - Thermal Joining Engineering, Rostock, Germany; 2 Saint-Gobain Coating Solutions S.A.S., Avignon, France; 3 Chair of Joining Technology, University of Rostock, Rostock, Germany

**Keywords:** alumina, atomizing gas, cord feedstock, electrical insulation, electrical resistivity measurements, flame spraying, scanning electron microscopy (SEM)

## Abstract

Besides conventional industrial demands, thermally sprayed coatings are increasingly used for innovative products. Such an application is the additive manufacturing of electrical components in automotive engineering. In particular, heating units are currently manufactured by a combination of various spray technologies. At present, simpler spraying processes like flame spraying are investigated with regard to their suitability as a future cost-effective alternative for fabricating isolating alumina coatings. In the present study, alumina cords were flame-sprayed using compressed air and argon as atomizing gases. The results demonstrate finely dispersed microstructures and a more regular and partially even higher surface and volume resistivity compared to past investigations in the literature as well as conventionally plasma-sprayed coatings despite a significantly reduced coating thickness. The content of alpha phase is clearly higher than for plasma-sprayed coatings, regardless of the atomizing gas used. Moreover, flame-sprayed coatings using argon reveal a higher resistivity in comparison to coatings sprayed with air. While the atomizing gas is found to mainly influence the ideal stand-off distance, the phase composition is not changed severely. In addition to the phase composition and kinematics, it can finally be concluded that humidity plays a major role in the coating properties.

## Introduction

In addition to applications in wear or corrosion protection, thermally sprayed coatings or coating composites are increasingly used in the electronics industry, e.g., in conductor tracks or integrated circuits (Ref 1, 2), due to their ability to adaptively adjust the final product properties (Ref 1, 3-5). Another field of application that is currently under intensive investigation is the additive production of electrical devices in automotive engineering. In particular, entire heaters for the vehicle interior are currently manufactured using a combination of different coating techniques due to a high degree of efficiency (Ref 6). An essential part for the proper function of such components is the ceramic isolating coating, which is mostly based on Al_2_O_3_ (Ref 1).

Today, alumina coatings in the industry are mainly produced by atmospheric plasma spraying (APS) (Ref 1, 7-9). However, it is common knowledge that thermally sprayed coatings of this material usually do not correspond to the same desired crystallographic modification as the bulk material. While sintered alumina thus mainly consists of the stable alpha phase, thermally sprayed coatings often experience process-induced transformations into several metastable phases like gamma, delta or theta (Ref 1, 9-12). Being of hygroscopic nature (Ref 1, 12), especially the metastable gamma phase has detrimental effects on the electrical resistivity of Al_2_O_3_ coatings, which is crucial for the planned application. Thus, several attempts have been made to reduce the amount of gamma phase inside alumina coatings by stabilization of the alpha phase, e.g., by addition other oxides such as Cr_2_O_3_ (Ref 1, 8, 9, 13-15). While the stabilizing effect is denied in some studies (Ref 9), others showed an increased amount of alpha phase (Ref 8, 13-15), which is partly dependent on the spraying process itself and thus the cooling rate of the particles as well as the Cr_2_O_3_ content. In other studies, a mixture of Al_2_O_3_ and TiO_2_ was flame-sprayed, showing that the phase composition partially correlated with oxy-fuel ratio. However, these coatings still were mainly composed of gamma phase (Ref 16).

In a study (Ref 11), the formation of metastable phases in thermally sprayed alumina coatings was described for the first time using nucleation theory. Thus, homogeneous nucleation as a result of supercooling results in gamma rather than alpha phase, since this phase requires a lower critical energy. According to this study (Ref 11), the phases are ultimately dependent on the thermal history of the particles as a result of melting as well as the kinetics of transformation, while the cooling rate is not considered to be particularly important. Therefore, the metastable gamma phase should be preserved mainly in small particles, while larger particles are more likely to transform towards the alpha phase (Ref 11). Furthermore, it is assumed that unmolten particles are more likely to serve as crystallization nuclei for the alpha phase due to comparable crystal orientation. The stabilization of the alpha phase by Cr_2_O_3_ described above is based on a reduction of the crystallization temperature for the gamma phase and a simultaneous increase in the transformation temperature for the alpha phase (Ref 8, 15). Using Cr_2_O_3_ on the other hand might result in decrease in electrical resistivity compared to pure alumina (Ref 17), which makes it probably rather unsuitable for the special application. Furthermore, it has been proved that epitaxial crystal growth is possible inside Al_2_O_3_ coatings at elevated temperatures and on single crystal alumina substrates, which means that adjusting substrate type and temperature can be used to control phase composition (Ref 18). Anyway, using temperatures of 900 °C is not an option for the desired components, since the substrates in this case consist of an aluminum alloy.

Another possibility to influence the phase composition and thus the coating properties as well as the deposition efficiency is the use of high-end or novel spray processes such as HVOF or suspension spraying by APS and/or HVOF (Ref 9, 12, 13, 17). In some papers, the final content of gamma remained nearly unchanged, when comparing HVOF- to APS-sprayed alumina coatings resulting in nearly unchanged or even slightly lower electrical resistivity (Ref 9). In other studies, however, HVOF-sprayed alumina showed a higher amount of alpha phase compared to their counterparts sprayed by APS. This led to increased electrical resistivity, especially at higher humidity (Ref 12). In addition, the use of suspensions in the S-HVOF process enables a further significant increase in the concentration of alpha phase as well as an improvement in the electrical isolation behavior (Ref 17). Another study with a newly developed technology named hybrid low-velocity oxy-fuel (hybrid-LVOF) revealed a finer microstructure and higher gamma to alpha ratio compared to conventional plasma spraying. Hence, this spray technology could be considered as alternative to plasma spraying (Ref 19). Since suspension spraying and hybrid-LVOF are still at the beginning of their industrialization and HVOF spraying is not significantly cheaper in direct comparison with plasma spraying, these processes are currently excluded as an economical alternative with regard to the planned industrial application.

In contrast to this, flame spraying is a cost-effective process in terms of acquisition, installation and operation with a high degree of efficiency (Ref 3, 7, 20), which in recent years has experienced some developments in the field of torch design and consumables (Ref 3, 21, 22). According to the literature, flame spraying using alumina rods often resulted in coarser coatings having a higher roughness and porosity compared to APS-sprayed coatings (Ref 23), while—as for APS—the gamma and delta phases were present inside the coatings (Ref 10). The use of cords on the other hand resulted in homogeneous coatings with porosity, microstructure and coating formation comparable to APS, likewise again revealing the presence of metastable phase (Ref 10, 18). However, the cited investigations were carried out with alumina rods and cords having relatively coarse powder fractions and using solely compressed air as atomizing gas. Recently, a new quality of cords providing finer microstructures is commercially available for flame spraying (Ref 22), which should enable the production of fine-structured coatings at low cost.

At the same time, it is widely known that e.g., in arc-spraying, the atomizing gases used can massively influence the coating formation and quality by significantly reducing exothermic reactions like oxide formation (Ref 2, 24). A change from compressed air to an inert gas thus could also promote reduction in hydroxides, which usually form during the spraying process of Al_2_O_3_ (Ref 8) by acting as a shroud and decreasing contact between the particles and the atmosphere. Apart from this, the atomizing gas used naturally also influences the transfer of momentum and heat from the flame to the particles, which tends to affect smaller particles more than large particles (Ref 1). Furthermore, the combustion of acetylene produces water, which has been proven to have a high content near the nozzle outlet for powder flame spraying (Ref 25). In addition to the presumably greater retention of humidity on smaller particles, water vapor also plays a major role as a potential catalyst or doping agent for the alpha phase. Various studies have highlighted the positive influence on the nucleation of this phase, which was explained partly by a non-diffusive transformation correlating with the formation of hydroxides (Ref 11, 26-28). It is therefore plausible that the atomizing gas used, in addition to the particle size itself, will play a decisive role in these phenomena.

In order to clarify the possible effects of different atomizing gases on the issues described above, argon and compressed air were used to produce thin flame-sprayed alumina coatings with a modern, finely structured cord with different thicknesses and varying stand-off distances. Afterward, these coatings were compared to plasma-sprayed reference coatings.

## Experimental Methods

### Materials and Spray Process

The flame spray experiments were carried out by using a MASTER JET® flame spray gun and the commercially available cord Flexicord ALUMINA SUPRA (Ø 4.75 mm and Al_2_O_3_ 99.7 %;) both supplied by Saint-Gobain Coating Solutions S.A.S., Avignon, France. For comparison purposes, plasma-sprayed reference coatings were produced by using a GTV Delta torch (GTV Verschleißschutz GmbH, Luckenbach, Germany). The substrates for the flame spray experiments were principally prepared and coated just like the reference coatings—i.e., sheets of an aluminum alloy (*t* = 3 mm) were roughened and then coated with a NiCr 80/20 bond coat. While the reference coatings were manufactured with a standard industrial parameter and thus resulted in a fixed coating thickness of 278 ± 14 µm, the flame-sprayed coatings were produced at different stand-off distances, and in different thicknesses (by adjusting the number of passes only), which can be found in Table 1 together with the resulting coating thickness and surface roughness Ra.

**Table 1:** Varying parameters of flame spraying experiments

Atomizing gas	Stand-off distance (SOD) in mm	Number of passes	Thickness in µm	Roughness Ra in µm
Compressed Air (C. Air)	70	8	52 ± 5	3.4 ± 0.1
		16	134 ± 6	3.5 ± 0.1
		25	240 ± 8	3.6 ± 0.1
	90	8	49 ± 5	3.5 ± 0.2
		16	91 ± 5	3.5 ± 0.1
		25	142 ± 6	3.5 ± 0.1
Argon (Ar)	70	8	70 ± 6	3.5 ± 0.2
		16	135 ± 4	3.5 ± 0.1
		25	212 ± 6	3.7 ± 0.2
	90	8	51 ± 6	3.6 ± 0.1
		16	106 ± 6	3.6 ± 0.2
		25	161 ± 5	3.7 ± 0.1
Plasma	Industrial parameter		278 ± 14	7.3 ± 0.4

Resulting coating thickness and surface roughness compared to the plasma-sprayed reference coatings

In order to be able to determine potentially relevant differences in the coating properties, the stand-off distances were chosen in a wider range around the ideal value according to the spec sheet (Ref 22), whereas this ideal distance should of course also depend on the atomizing gas. The other parameters were kept constant and are summarized in Table 2.

**Table 2:** Constant parameters of the flame spraying experiments

Acetylene pressure in MPa	0.12
Oxygen pressure in MPa	0.4
Atomizing gas pressure (C. Air / Ar) in MPa	0.4
Robot traverse speed in mm/s	500
Spraying pattern	Meander-shaped

For each variation, three samples were coated. If the trends of the different coating thicknesses are similar, only the coatings with 8 passes are shown; otherwise, the results of all coatings are shown, of course.

### Surface Roughness

The tactile Hommel-Etamic T8000 and the corresponding EVOVIS software (both JENOPTIK AG, Jena, Germany) were used to determine the surface roughness in the as-sprayed state. The cut-off wavelength *λc* was selected to be 2.5 mm. The surfaces were measured in measuring directions of 45°, 90° and 135° offset to the sample width. The characteristic values of the arithmetic mean roughness Ra were then calculated as mean values from these directions plus from all manufactured specimens per variation.

### Thermographic Temperature Measurements

Surface temperatures of the specimens during coating build-up were measured by using a thermographic camera VarioCAM hr 675s and the software IRBIS3 professional (both InfraTec GmbH, Dresden, Germany). A frame rate of at least 5 fps and an emissivity of 0.78 (corresponds to sintered Al_2_O_3_) were used for recording the spraying processes. Since the temperatures during the coating process are considered for comparative purposes, histograms were computed by an in-house MATLAB routine, resulting in temperature distributions.

### Electrical Properties

**Fig. 1:**
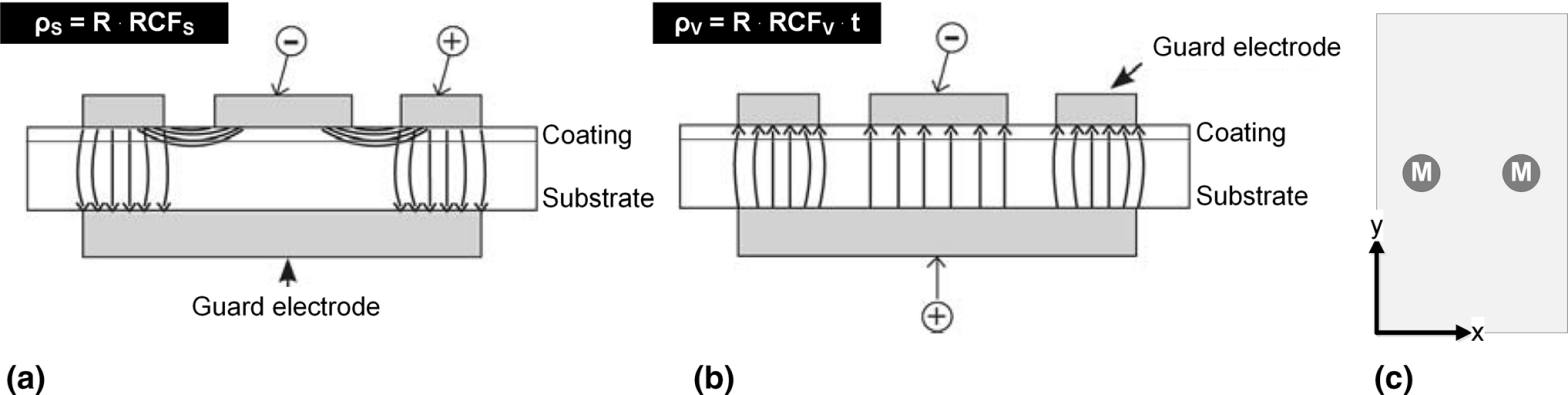
Principal of the ring electrode method for measuring (a) the specific surface resistivity and (b) the specific volume resistivity. Both are influenced by correction factors RCF (compensate for differences in electrical energy), while only the volume resistivity is affected by the specimen thickness t. (c) Measuring scheme in top view

In general, the specific surface resistivity of the coatings was determined by the ring electrode method, which is illustrated schematically in Fig. 1(a). For this purpose, a computer-aided Hiresta UX MCP-HT800 system (Mitsubishi Chemical Analytech Co. LTD, Kanagawa, Japan; optimum voltage adapted to each specimen in a range from 50 to 1000 V), with 2 measuring points for every specimen recording 7 values each was used, see Fig. 1(c).

In order to determine the influence of the expected hygroscopic behavior of the coatings, the surface resistivity was measured in different states. Some specimens were measured in the as-sprayed state directly after the spraying process as well as after 7 and 49 days, respectively, without any further treatment. The time intervals of the measurements did not follow any given pattern, but correlated rather with the other investigations performed. Moreover, some of the specimens were first heat-treated at different temperatures and measured subsequently, while others again were heat-treated and stored at room temperature before the measurements were carried out.

In addition, the volume resistivity was measured for some specimens in various states (as-sprayed and exposed to room temperature; heat-treated and stored at room temperature) to obtain a comparable characteristic material parameter with regard to both spray processes, which is shown in Fig. 1(b) as a schematic diagram. For this objective, it is necessary to use the total thickness of the corresponding specimen, which is the sum of substrate thickness, bond coat and ceramic coating. Furthermore, the insulating base Resi Table MCP-ST03 (Mitsubishi Chemical Analytech Co. LTD, Kanagawa, Japan) is used as ground to reduce impact of leakage currents and external influences on the results. Measuring device and procedure correspond to the ones already described above, except for the latest measurements (342 days after spraying). These ones were carried out by the help of an own newly developed computer routine, which allows to determine the optimal measurement range more precisely by scanning different parameters (range, voltage). This enables more accurate measurements and less scattering of the results.

### Microstructural Analyses

For the purpose of microstructural analyses, the specimens were first cold-mounted (two-phase system: liquid hardener and powder resin) and gradually ground and polished (6 µm, 3 µm suspensions, finally oxide polish). Coating thickness was investigated by using an optical microscope (OM) Leica DM6000M (Leica Microsystems GmbH, Wetzlar, Germany) and the software tool ImageAccess (Imagic Bildverarbeitung AG, Glattbrugg, Switzerland) recording 3 times 7 measured values. Further, a scanning electron microscope (SEM) JEOL JSM-IT100 (JEOL Germany GmbH, Freising, Germany; magnification 1000x, acceleration voltage 10 kV, backscatter detector and low vacuum mode) and the software ImageJ (National Institutes of Health, USA; in region of interest using Despeckle filter and binarization via histogram) were used for examining the porosity of the coatings at 3 areas inside each specimen. Moreover, representative analyses regarding the morphology of the coatings and the used cord were carried out by using the same SEM at different magnifications (500× and 2000×). In addition, the particle size distribution of the cord was determined at 6 measuring points in total (1 in the center and 1 at the edge of the cord in 3 specimens of the cord) using the same equipment and software as already described above, but at different magnifications (100×, 250× and 500×). After determination of the average Feret diameter, histograms were plotted and *d*_10_, *d*_50_ and *d*_90_ of the alumina particles were identified

### XRD Phase Analysis

X-ray diffraction (XRD) measurements were performed by using a D8 Discover with LYNXEYE detector (Bruker AXS GmbH, Karlsruhe, Germany; acceleration voltage of 40 kV and arc current of 40 mA) with Cu-Kα radiation at a wavelength of 0.154 nm. Experiments were carried out with a primary and secondary orifices of 2.5° and differential orifice of 0.34°. Resulting XRD patterns were evaluated quantitatively using Rietvield method and TOPAS software (also Bruker AXS GmbH). Phase analyses have been performed for the flame-sprayed coatings with 8 passes and the plasma-sprayed reference, while none of these samples underwent heat treatment. Therefore, the phase composition should correspond as far as possible to the as-sprayed condition, although these investigations were not carried out until about a year later.

## Results

### Examination of the Cord

**Fig. 2:**
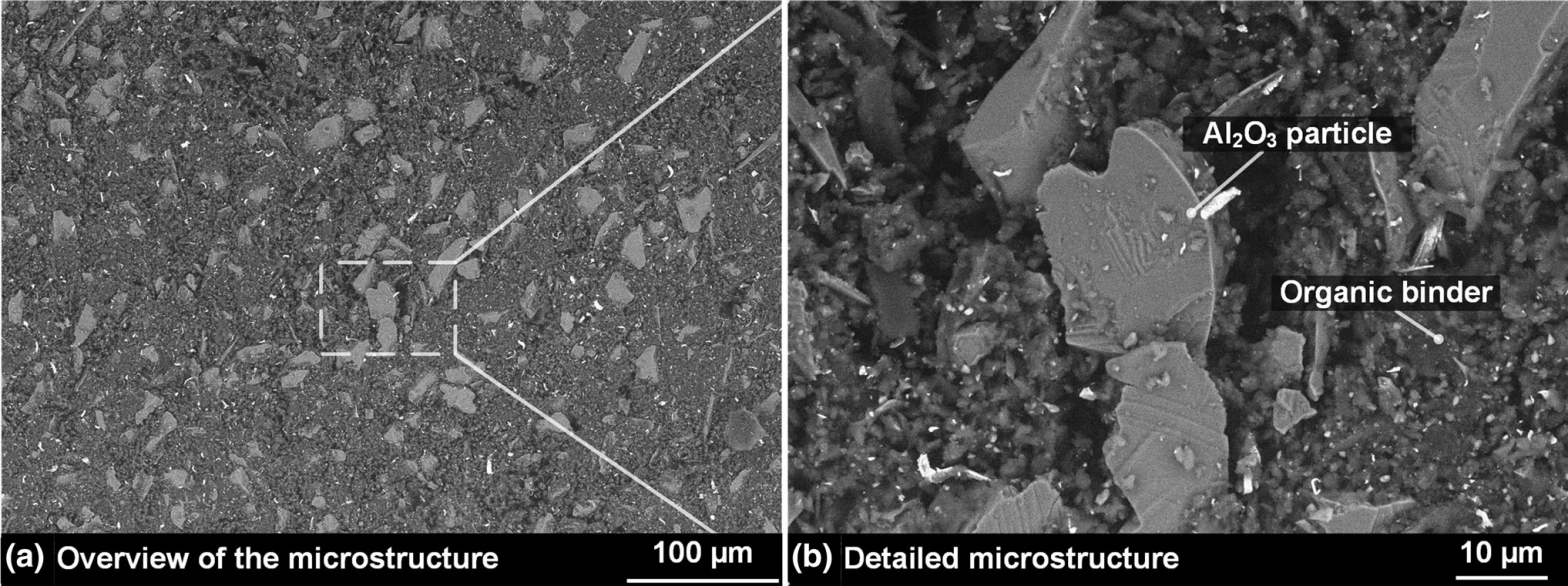
Microstructure of the cord in SEM using backscatter detector and LV-Mode at 10 kV with (a) an overview and (b) a detailed view showing the ceramic particles and the organic binder matrix

Figure 2(a) shows the typical microstructure overview of the used cord, while the detailed microstructure can be viewed in Fig. 2(b). It can be seen that it has a fine microstructure with relatively small Al_2_O_3_ particles produced by melting. These particles are embedded and evenly distributed inside the soft organic binder matrix.

**Fig. 3:**
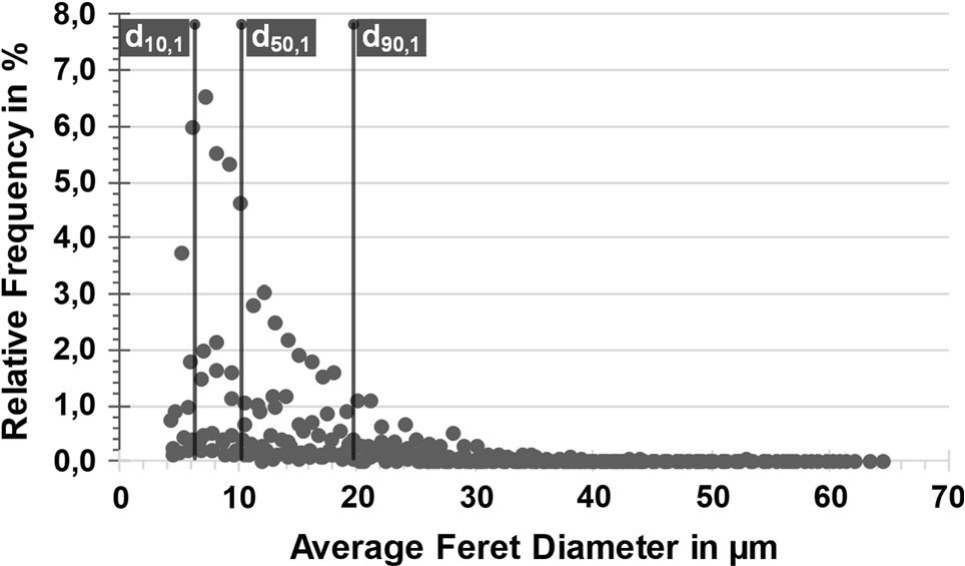
Average Feret diameter for measuring point 1; exemplary representation for all measuring points

The analyses of the particle size distribution proved the representative description above. An exemplary representation for one of the examined measuring points can be found in Fig. 3.

The average Feret diameter of the particles *d*_50_ is 10.2 ± 0.5 µm, while *d*_10_ and *d*_90_ correspond to 6.1 ± 0.1 µm and 19.7 ± 1.2 µm, respectively. Moreover, together with the homogeneous distribution throughout the cord, the determined powder fraction should be a sufficient foundation for the desired fine microstructure of the coatings.

### Microstructural Investigation of the Coatings and Surface Quality

In Table 1, the coating thicknesses for the thinnest coatings reveal homogeneous values in a narrow regime with low standard deviations, except for the coating with a SOD of 70 mm and Ar. Coatings sprayed with 16 or 25 passes, however, show different trends. For these coatings, a higher SOD results in a lower coating thickness, whereby the values for comparative passes are more alike. The use of Ar on the other hand mostly increases coating thickness, especially for a SOD of 90 mm. Compared to the plasma-sprayed reference, none of the flame-sprayed parameter sets achieves a similar coating thickness, which may also be related to the fact that SOD, and thus deposition efficiency, are not regarded as ideal due to the intended parameter selection. Even considering the ranges of standard deviations, the thickest flame-sprayed coating is still about 15% thinner than the reference.

**Fig. 4:**
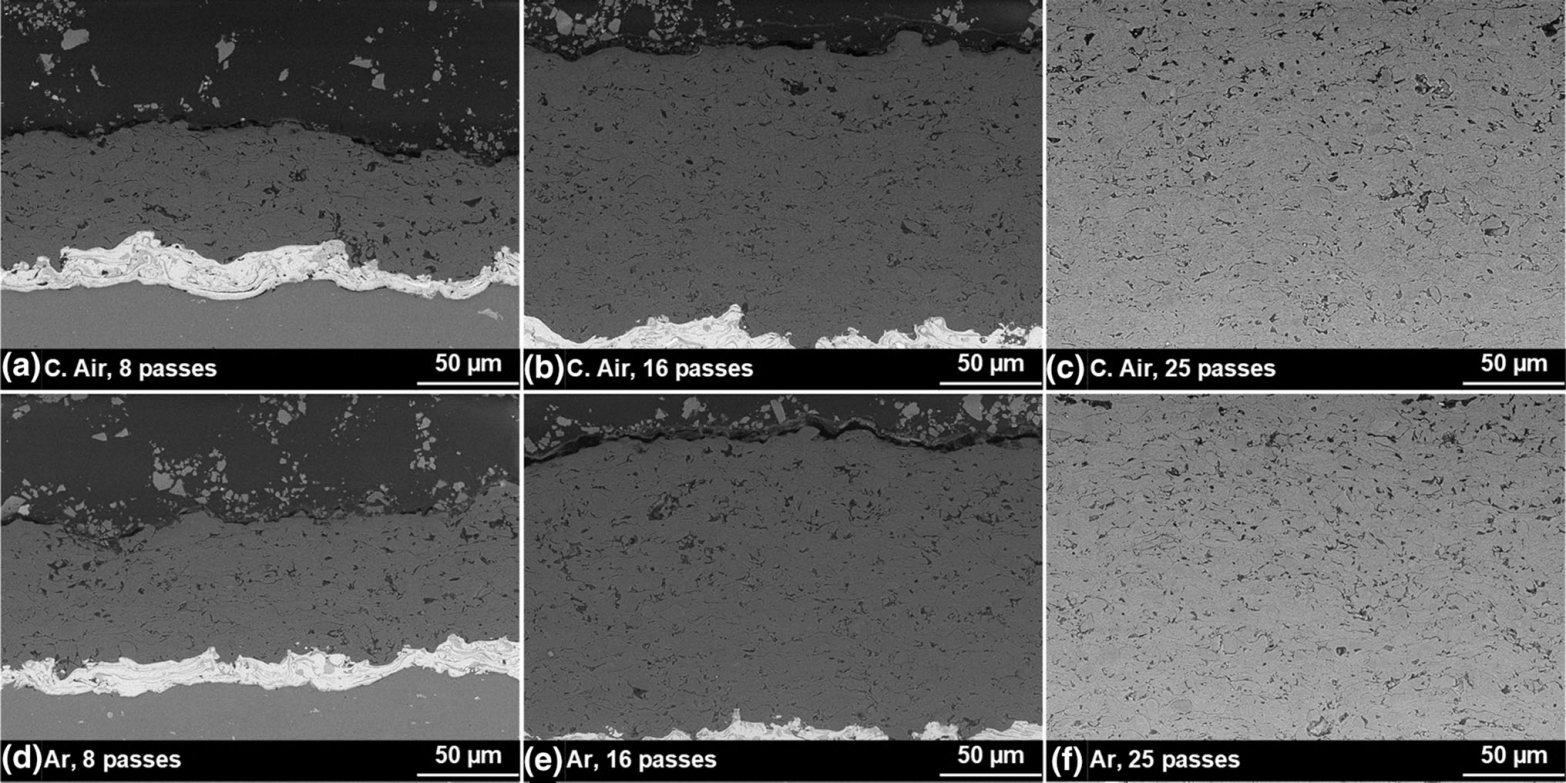
Overview of the microstructures regarding the coatings sprayed with compressed air (top) and argon (bottom) at a SOD of 70 mm for (a) and (d) 8 passes, (b) and (e) 16 passes, (c) and (f) 25 passes

A brief overview concerning the typical microstructures for all numbers of passes and both gases can be found in Fig. 4.

The open porosity grows towards the coating surface with increasing thickness, thus revealing a graded structure for more than 8 passes. In principle, the trend applies to a SOD of 90 mm (not displayed) too. However, this phenomenon is not as pronounced for coatings sprayed with Ar as for coatings sprayed with C. Air. Instead, the overall porosity is distributed finer than for C. Air, while at first sight it appears to be of the same amount. In addition, the thicker (more than 8 passes, not displayed) coatings with a SOD of 90 mm show an even better distribution of porosity and a denser coating structure near the substrate in direct comparison.

**Fig. 5:**
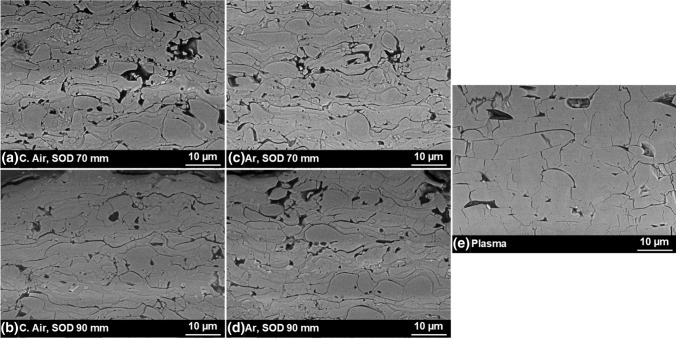
Detailed microstructure of the flame-sprayed coatings made in 8 passes, in (a) and (b) using compressed air and in (c) and (d) with argon as atomizing gas. The typical microstructure of the plasma-sprayed reference coatings is displayed in (e)

The detailed microstructures for coatings sprayed with 8 passes are compared with each other and the plasma-sprayed coatings in Fig. 5.

First, all of the flame-sprayed coatings reveal a smaller particle size than the reference coatings and thus finer structure with a changed morphology of the splats. While the plasma-sprayed coatings are typically characterized by large, plate-like cubic particles, the flame-sprayed coatings show a mix of lamellar and globular particles. At the same time, the porosity seems to be in a similar range, but distributed differently.

When comparing the specimens regarding the atomizing gases, it is apparent that the use of Ar results in different effects, dependent on the SOD. While for a SOD of 70 mm, even smaller splats and a finer microstructure including less porosity can be observed, Ar for an SOD of 90 mm apparently leads to slightly higher porosity. Moreover, the pore content seems to be less clustered and smaller compared to C. Air, compare Fig. 5(c) and (d) with (a) and (b). In the case of SOD, a higher value leads to slightly bigger particles and a more homogeneous distribution of pores in the case of C. Air, while it clusters again considering Ar.

**Fig. 6:**
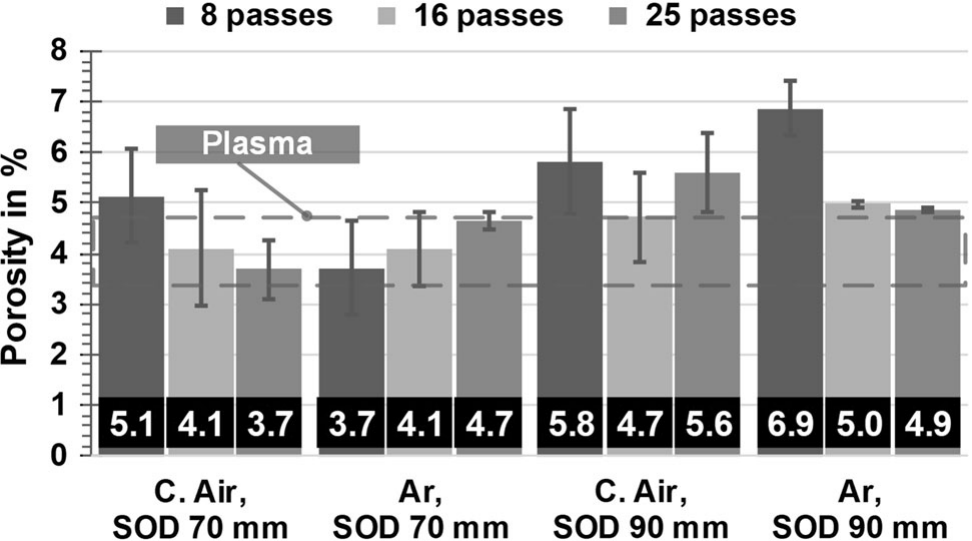
Porosity of the flame-sprayed coatings in comparison with each other and the plasma-sprayed reference coatings

Quantitative porosity measurements in Fig. 6 generally confirm the observations made before.

The measurements reveal different trends, especially when comparing different SOD. For a SOD of 70 mm, the porosity is mostly reduced by the use of Ar in comparison with C. Air. For both gases, these specimens are also in reach of the reference coatings, albeit having far more particle interfaces due to the reduced splat size. Yet, the standard deviation remains nearly unchanged. The observed trend for a SOD of 90 mm is different, since the porosity even increases by the change of gas from C. Air to Ar for some specimens. In terms of percentage, however, this increase is less than the reduction in the case of a SOD of 70 mm. Furthermore, the scattering of the values is reduced by the use of Ar. In addition, with the exception of Ar and a SOD of 70 mm, the porosity tends to decrease as the number of passes and thus the coating thickness increases. Nevertheless, these differences are comparatively small, considering the scattering width. Comparing the specimens by SOD, it is obvious that the results are better for 70 mm.

In the case of surface quality, the coatings do not experience real differences. All the flame-sprayed variations reveal similar values from 3.4 to 3.7 µm having no observable trend, see Table 1. The comparison with the plasma-sprayed reference, however, shows a finer surface, which is about factor 2 lower.

### Temperature Regime

**Fig. 7:**
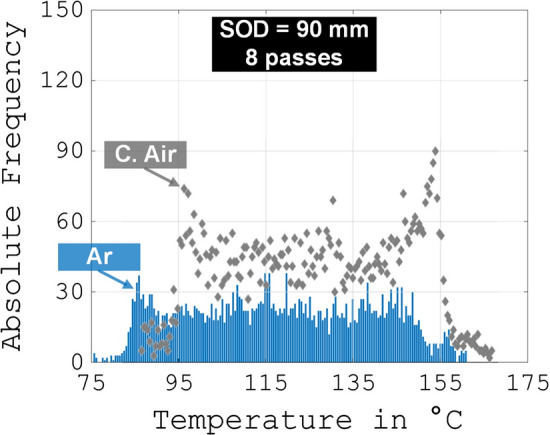
Temperature distribution, representatively for SOD = 90 mm and 8 passes. The blue bars represent the histogram regarding the average surface temperature during spraying for the coating sprayed with argon. The grey rhombs indicate the histogram of the corresponding coating sprayed with air

The process temperatures are displayed exemplarily in Fig. 7.

The shapes of the histograms differ substantially for both gases. First, the overall maximum temperature for C. Air (167 °C) is higher than for Ar (161 °C). Moreover, the frequency of temperature peaks near the temperature with the highest absolute frequency is much higher for C. Air, e.g., near 154 °C (frequency of 90 for C. Air compared to 38 for Ar). It is also noticeable that a further accumulation of temperature peaks occurs at the left end of the temperature distribution of C. Air. Neither of these observations applies to the temperature distribution of Ar. On the contrary, the distribution is very homogeneous over the entire spectrum and shows less scattering. Furthermore, the histogram for Ar is shifted to the left towards cooler temperatures. These facts are also confirmed by the temperature with the highest frequency. In principle, these findings also hold true for the samples with a SOD of 70 mm, despite the fact that the temperatures are generally, as expected, slightly higher. The same accounts for the thicker specimens, while as expected, the absolute frequencies rise in general as do the temperatures slightly.

### Specific Surface Resistivity

**Fig. 8:**
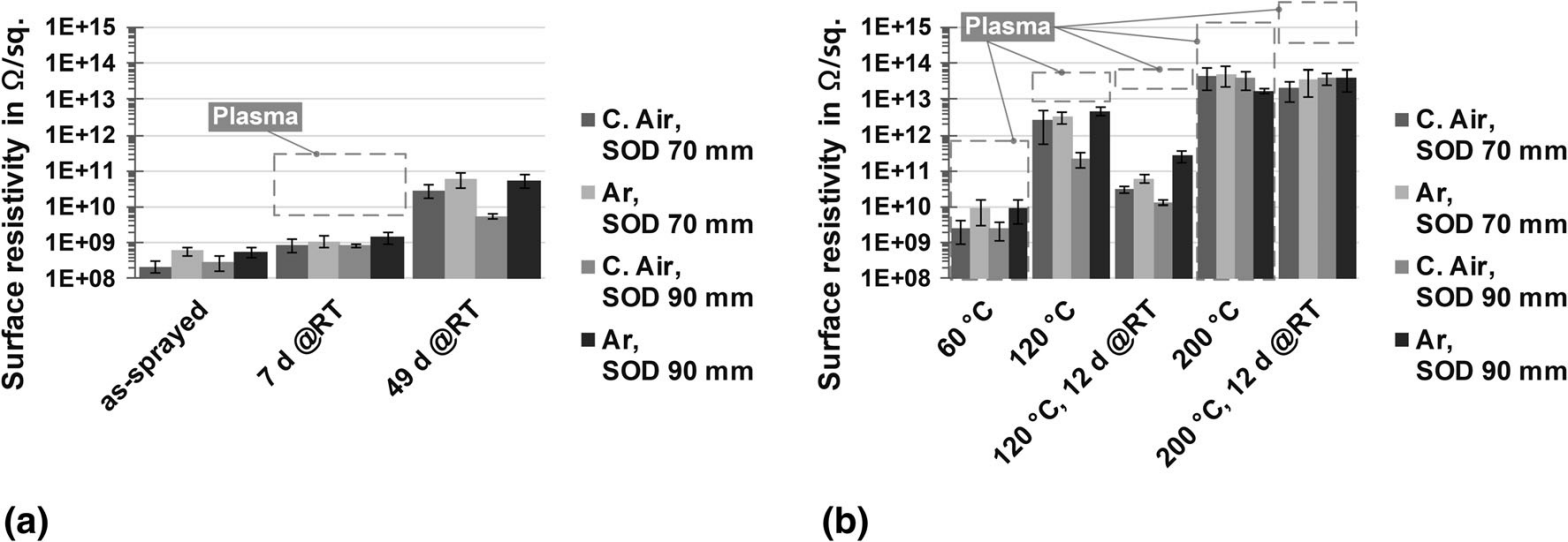
Specific surface resistivity in Ω/sq of the flame-sprayed coatings made in 8 passes. The coatings in the as-sprayed state, i.e., initial, after 7 days and 49 days at room temperature, are represented in (a). In (b), the results of the heat-treated (measured directly after cooling) plus heat-treated and stored (12 days at room temperature each time) specimens are displayed. If available, a gray line indicates values of the plasma-sprayed reference coatings

The results of the specific surface resistivity measurements in different conditions and for 8 passes can be found in Fig. 8.

At first, it can be seen that the values in the as-sprayed state in Fig. 8(a) are in the order of magnitudes between 10^8^ and 10^9^ Ω/sq., with the Ar-sprayed coatings showing higher values and less variations for both SOD. In addition, it is clearly visible that the resistivity increases with increasing exposure to ambient temperature, instead of decreasing as anticipated. While the increase is still moderate after 7 days of storage, the values have grown by a factor of about 100 after 49 days. For both storage periods, it is still true that the coatings sprayed with Ar have higher levels than those produced with C. Air. Nevertheless, after 7 days, the surface resistivity is still not at the same level as the plasma-sprayed reference coatings.

Different trends can be observed for the heat-treated samples in Fig. 8(b). The values for the coatings sprayed using Ar are generally higher in almost all cases than for the comparable coatings with C. Air. However, the values for samples heat-treated at 60 °C remain below those of the samples in Fig. 8(a), which just have been stored for 49 days. In comparison, the samples at 120 and 200 °C show a much more significant increase in resistivity up to a factor of about 10^5^ compared to the initial values. The subsequent exposure to room temperature, however, revealed different effects in the samples at these two temperatures. While the resistivity decreases in the case of 120 °C significantly due to storage (by a factor of about 100), it remains almost constant regarding 200 °C in total. In this context, the comparatively low scattering of the flame-sprayed coatings compared to the reference coatings is also strikingly visible, although the values are only in partially similar ranges at 60 and 200 °C. However, this estimation is difficult due to the very high scattering of the plasma-sprayed coatings. In general, the resistivity increases with a higher number of passes—for 16 passes especially for the samples heat-treated at 200 °C. The highest values are found for 25 passes. Overall, it should be noted that the surface resistivity as a means of characterizing the coatings themselves is not satisfactory, although it does provide an impression of the influence of environmental conditions on the electrical properties.

### Specific Volume Resistivity

**Fig. 9:**
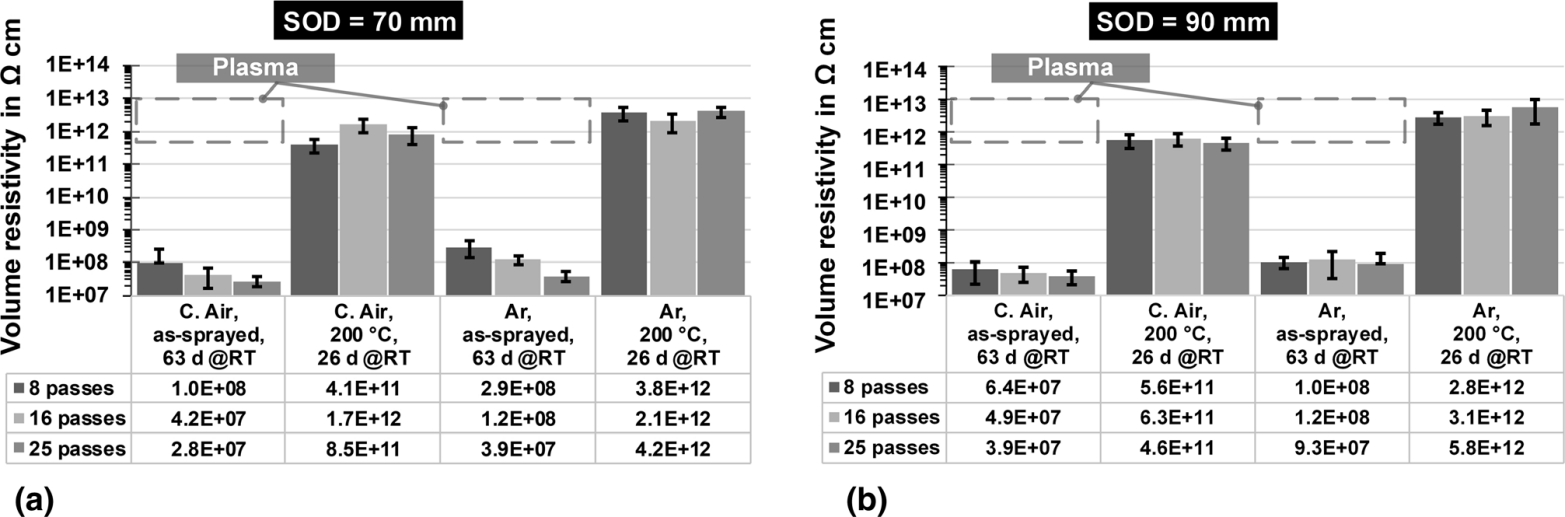
Specific volume resistivity in Ω cm of the flame-sprayed coatings with 8, 16 and 25 passes showing (a) the coatings with SOD of 70 mm, while (b) displays the specimens with SOD of 90 mm. The three bars on the left side each represent the untreated samples stored at room temperature for 63 days. The bars to the right represent samples that were heat-treated at 200 °C and then stored at room temperature for 26 days. In addition, the effect of the atomizing gases is shown in both parts of the figure. Moreover, the average value is listed in the table below. If available, a gray line indicates values of the plasma-sprayed reference coatings

The specific volume resistivity measurements are shown in Fig. 9.

In the first instance, the results are far more homogeneous than the surface resistivity measurements and exhibit less variation, which in part can be attributed to the measurement method itself. This in part also accounts for the reference coatings. Regarding the two atomizing gases, coatings sprayed with Ar have higher values and a lower scattering than the comparable coatings sprayed using C. Air for both SOD and conditions. It can also be noted that the thicker coatings tend to show a decrease in resistivity when untreated, while the C. Air coatings have a lower base level. In general, the values vary less for Ar and are almost independent of coating thickness. Furthermore, varying trends can be observed considering the influence of SOD, compare Fig. 9(a) and (b). While for 8 passes a SOD of 70 mm is more suitable for producing coatings of high resistivity, a SOD of 90 mm is more advantageous for the thicker coatings. An exception to this are coatings that have been sprayed with C. Air, heat-treated at 200 °C and then stored, see Fig. 9(a), where the trend is exactly the opposite.

While the flame-sprayed coatings have values in the order of magnitude of about 10^7^ to 10^8^ Ω cm in the as-sprayed state, the values for the plasma-sprayed coatings are significantly higher. However, it should be noted that these values were more difficult to measure due to strong fluctuations in the measurement process. This led to fewer measured data and a higher variation compared to the flame-sprayed coatings. After heat treatment and subsequent exposure to room temperature, the resistivity of the flame-sprayed coatings was even in the range of the untreated reference coatings, with more homogeneous values at the same time. A final resistivity of about 10^11^ to 10^12^ Ω cm in the heat-treated and stored specimens was not reported in the literature so far. On the contrary, the coatings in the as-sprayed condition were more in line with the usual values in the literature (Ref 1). Finally, considering the comparison in Table 3, the resistivity for the untreated flame-sprayed specimens increases even further over time, essentially by about one order of magnitude. In contrast, the resistivity of the plasma-sprayed reference is gradually decreasing and continues to show a strongly enhanced scattering in comparison with the flame-sprayed coatings. After about one year of storage, it is therefore not yet clear where the final resistivity value will be for the flame-sprayed coatings. While the improved measurement methodology for the flame-sprayed coatings is evident in reduced scattering of results in percentage terms, the same is not true for the plasma-sprayed reference. This confirms the previous observations on the overall higher scattering width of these coatings.

**Table 3:** Quantitative phase estimation by XRD and long-term specific volume resistivity

Specimen	Quantitative estimation by XRD		Specific volume resistivity in Ω cm	
	α in %	γ in %	As-sprayed, 63 days @RT	As-sprayed, 342 days @RT
C. Air, SOD 70 mm	41.02	58.98	1.0 (± 1.4) 10^8^	1.7 (± 0.7) 10^9^
C. Air, SOD 90 mm	37.76	62.25	6.4 (± 4.2) 10^7^	3.3 (± 0.9) 10^8^
Ar, SOD 70 mm	41.55	58.45	2.9 (± 1.5) 10^8^	1.9 (± 1.0) 10^9^
Ar, SOD 90 mm	36.35	63.65	1.0 (± 0.4) 10^8^	3.8 (± 0.4) 10^9^
Plasma	10.57	89.43	6.4 (± 6.0) 10^12^	1.6 (± 1.8) 10^12^

### Phase Composition

**Fig. 10:**
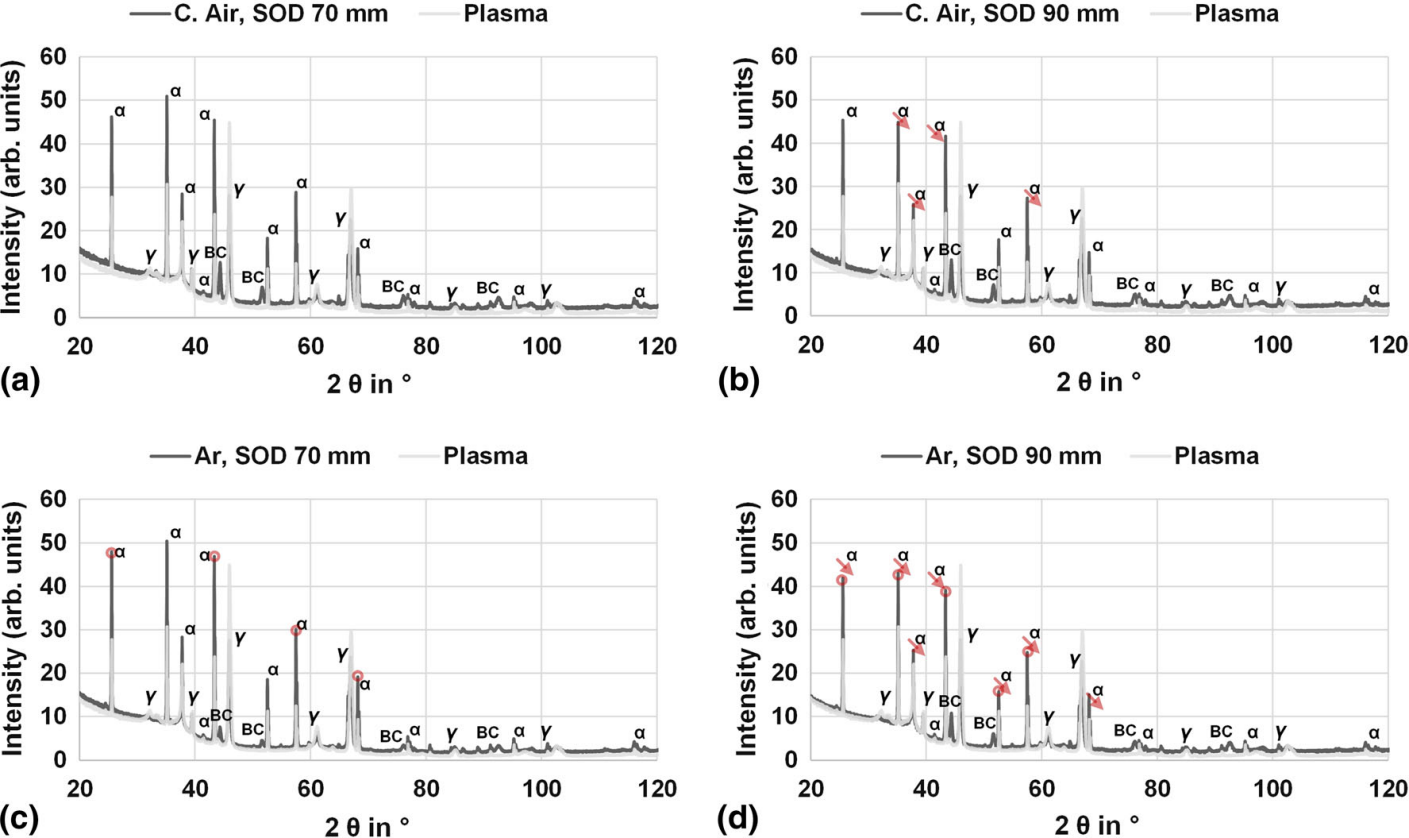
XRD patterns of the flame-sprayed coatings with C. Air at a SOD of (a) 70 mm and (b) 90 mm as well as with Ar at a SOD of (c) 70 mm and (d) 90 mm. The plasma-sprayed reference coating is indicated as a light grey line. The labels on the peaks show the corresponding phases, where α indicates the alpha phase, y represents the gamma phase and BC stands for the NiCr80/20. The red arrows show a reduction of the peaks from left to right, i.e., for the respective increase in the spray distance. The red circles, on the other hand, show a change (positive and negative) for identical spraying distance, i.e., from top to bottom. While the arrows thus describe the influence of the SOD, the circles are a representation of the effect of the atomizing gas

The XRD patterns of the flame-sprayed coatings with 8 passes and the plasma-sprayed reference coating can be found in Fig. 10.

First, all flame-sprayed samples show similar patterns with peaks in mostly comparable regions, consisting of alpha and gamma phase predominantly. Due to the low coating thickness, even the NiCr 80/20 is detectable.

The change in atomizing gas for identical SOD, i.e., from Fig. 10(a) to (c) or from Fig. 10(b) to (d) (indicated by red dots), has only a minor effect on the composition in the form of the alpha phase. In some cases, increases can be observed in addition to slight decreases. This small trend is somewhat more pronounced for a SOD of 90 mm than for 70 mm. The gamma phase content, on the other hand, can be considered independent of the atomizing gas.

In contrast, for different SOD with identical atomizing gas, i.e., from Fig. 10(a) to (b) or Fig. 10(c) to (d) (indicated by the red arrows), a clearer trend can be seen. The increase in SOD has a consistently reducing effect on the alpha phase, although this trend is visibly more pronounced when Ar is used. Here, the gamma phase peaks remain unaffected too.

However, the plasma-sprayed reference shows that the peaks differ significantly from the flame-sprayed coatings having much smaller peak values of alpha and higher maxima of gamma, while the NiCr 80/20 bond coat was not detectable.

The above-mentioned observations made in Fig. 10 are supported by Table 3, showing the results of the quantitative evaluation of the XRD measurements.

In general, it can be stated that the influence of the atomizing gas is less than that of the SOD involved in the formation of the alpha phase. Furthermore it can be seen that the plasma sprayed coatings have a significantly lower proportion of alpha phase and a higher proportion of gamma phase. However, it should be considered that no reference material was available for the evaluation. While this is less problematic for the flame-sprayed coatings, the plasma sprayed reference showed some peak widening, possibly due to partially amorphous or nanocrystalline fractions.

## Discussion

At first, it was completely unclear whether such partially thin coatings could provide a satisfactory resistivity. This becomes particularly apparent when considering the differences in the average coating thicknesses; the plasma-sprayed reference coatings are on average about five times thicker than the thinnest flame-sprayed coatings, see Table 1, while the differences are less for coatings made in 16 or 25 passes. It was expected that due to the nature of thermally sprayed alumina coatings and the accompanying comprehensive transformation from alpha to gamma phase, the coatings are rather hygroscopic. This would lead to an increase in humidity over time inside the coatings and thus a reduction in resistivity, as is known from conventional plasma-sprayed coatings (Ref 1, 8, 12). In fact, similar to the low thickness, this seems to play a minor role. Instead, a superposition of at least three effects seems to determine the resistive properties of the coatings.

The first of these is the humidity absorption of the particles during the flight from the gun until they hit the substrate, the application pass by pass and in the subsequent cooling process. Since the droplets in flame spraying—also due to the fine powder fraction of the cord, compare section “Examination of the Cord”—are significantly smaller than for plasma spraying, they have a higher specific surface area and cool down faster. This is confirmed by Fig. 5 in section “Microstructural Investigation of the Coatings and Surface Quality” by the microstructure itself as well as the lower surface roughness in comparison with the plasma-sprayed reference coatings, see Table 1. Thus, the particles in flame spraying bind more humidity during the flight and completion of the spray pass and during the cooling process. This humidity arises on the hand from the atmosphere itself and is thus partly dependent on the ambient conditions at the location. Additionally, it is known that flame combustion of acetylene with oxygen produces water (Ref 7), which has been proven to even exist near nozzle outlet for powder flame spraying (Ref 25). Hence, another source of humidity could be the combustion process itself. Compared to the other factors that affect the total humidity absorbed, though, this influence can probably be classified as rather small. The humidity, which therefore consists of several factors, is entrapped in the residual coating porosity, which partially explains the low specific surface resistivity in the as-sprayed state, see Figure 8(a) and 9. Nevertheless, the values considering the volume resistivity are less influenced and in good agreement with range reported in the literature (Ref 1, 9).

However, instead of an expected decline, an increase in the surface resistivity with increasing storage becomes apparent. The samples with the longest exposure time even show values higher than after heat treatment at 60 °C, compare section “Specific Surface Resistivity.” These facts lead to the conclusion that the initially retained humidity can evaporate over time up to a certain point. The same effects were visible for the coatings with 16 and 25 passes possibly due to the graded microstructure, see Fig. 4. This observation correlates with the observations in the XRD measurements in Fig. 10 and Table 3 revealing less gamma phase and more alpha phase in the flame-sprayed coatings. Considering the heat-treated specimens in sections “Specific Surface Resistivity” and “Specific Volume Resistivity,” it became clear that a significant increase in surface (and volume) resistivity generally occurred at the higher temperatures. However, the subsequent exposure to the environment had different effects. The fact that the resistivity in this particular case remains almost stable only at the higher temperature of 200 °C indicates, in accordance with (Ref 8) that from this point on some aluminum hydroxides might finally be dissolved theoretically. These also arise from the chemical reactions of the particles with dissolved water vapor described above and affect coating properties, but should be considered separately from the pure trapped humidity. This thesis is further supported by the fact that both surface and volume resistance were higher for coatings sprayed with Ar, see Figure 8(a) and 9. The use of Argon to propel the particles diminished the influence of the atmosphere and thus reduced both the humidity itself and the formation of corresponding hydroxides, while the share of alpha and gamma phase is almost the same, when considering identical SOD, see section “Phase composition.” Another fact that could at least indirectly confirm this is the more homogeneous temperature distribution shifted to cooler temperatures when using Ar instead of C. Air, see section “Temperature Regime.” This indicates a lower degree of exothermic reactions such as the formation of hydroxides.

Moreover, the flame-sprayed coatings contain more alpha phase and less gamma phase than conventionally sprayed coatings, compare Fig. 10 and Table 3, whereas the latter was further confirmed by evaluating resistivity behavior over time. The volume resistivity for untreated flame-sprayed specimens increased even after about a year of storage, while the value for the plasma-sprayed decreased, see Table 3. In addition, the use of Ar results in change of heat and momentum transfer from the flame to the particles due to the lower thermal conductivity compared to pressurized air. Together with the small particle size that might play a role in the thermal history of the particles too and hence coating / phase formation, since smaller particles adapt more to the properties (velocity, heat) of the gas jet than larger particles (Ref 1). Thus, the particle size can also be considered as a possible explanation for different microstructural trends in porosity for the varying SOD described in section “Microstructural Investigation of the Coatings and Surface Quality.” This is also plausible, since the influence of atomizing gas on phase composition is negligible, while the SOD clearly influences it. The graded structure for the thicker coatings (16 and 25 passes) might be attributed to an increasing plastic deformation of the lower, already deposited and still warm layers by the newly impinging particles. The higher SOD here presumably leads to a greater influence of large particles, since small particles reach the substrate only in low quantities at this spraying distance.

Also, according to the literature, non-molten particles can additionally serve as nuclei for the alpha phase (Ref 8). A further possibility which must be considered with regard to phase transformations is the catalyzing effect of water vapor on the formation of the alpha phase (Ref 11, 26-28). This effect is certainly also influenced by the use of Ar instead of C. Air, although it is very difficult to measure and maybe partly counteracts the shroud mechanism to a certain extent. The aforementioned facts are plausible due to the microstructure of the cord used and the coatings see sections “Examination of the Cord” and “Microstructural Investigation of the Coatings and Surface Quality” and the measurements by XRD.

In general, it can be noted that the flame-sprayed coatings display less scattering than the plasma-sprayed reference coatings, which in part is probably the result of the finer microstructure and the resulting mechanisms mentioned above, which is particularly evident in the resistivity. However, this might also partially be attributed to the higher surface roughness of the plasma-sprayed coatings, being detrimental to the resistivity measurements. Moreover, the coatings sprayed with Ar are superior to the samples manufactured with C. Air. In the case of SOD, 70 mm is the better choice for thin coatings with C. Air, while a SOD of 90 mm produces higher resistivity for coatings sprayed with a higher number of passes and thin coatings with Ar. As the residual porosity is comparable to the reference coatings, this property does not seem to have much influence on the other coating properties, except for the retention of humidity.

## Summary and Outlook

In this study, flame-sprayed coatings were produced using argon and compressed air as atomizing gases and compared with plasma-sprayed coatings with respect to their coating properties. In general, these coatings may potentially serve as an alternative to plasma spraying as the following properties could be determined.

1. Their specific resistivity was found to be at the level of much thicker plasma-sprayed coatings when subjected to thermal post-treatment. However, if they remained untreated and were only stored, the resistivity also increased over a longer period of time, although not yet to the level of the plasma sprayed reference. In contrast, the reference always showed a greater scattering of the measured values and a decrease in resistivity over time.
2. The flame-sprayed coatings showed a comparable amount of porosity to the plasma-sprayed reference, while a much finer microstructure was detected. Moreover, significantly higher contents of alpha phase and a severe reduction of the gamma phase were observed. These findings are in good agreement with the observations regarding the resistivity.
3. The influence of atomizing gas is of more indirect nature in reducing the contact with the atmosphere and changing coating formation process, e.g., by affecting ideal stand-off distance. Thus, the results, e.g., in terms of resistivity, are also influenced by the variation of the stand-off distance. This finding is in good agreement with the temperature measurements revealing less exothermic reactions and reduced temperatures for Ar in compared to C. Air. In contrast, the atomizing gas has no effect on the phase composition under the same spraying conditions.
  However, due to the postulated mechanisms behind these observations, it must be assumed that the use of the coatings without heat-treatment will be difficult. The mechanisms that decisively determine the coating properties, especially the electrical properties, are briefly summarized in the following:
  A) The entrapped humidity inside the residual porosity, which is probably absorbed to a large extent during the particle flight and the layer-by-layer coating and possibly in rather small amounts during the combustion itself.
  B) Phase composition, i.e., different contents of alpha and gamma, of the final coating including the influence of water vapor and humidity over time.
  C) The possible formation of aluminum hydroxides due to chemical reactions, which must be considered a theoretical assumption at this point, since detection is difficult.

While (A) can be influenced by the exposure time and therefore will not be a problem at some point and (B) seems to be less critical than assumed so far, the complete dissolution of hydroxides in (C) and the complete evaporation of the water of (B) by heat treatment is difficult to justify economically. In addition, the phase composition is strongly dependent on various process parameters and in turn influences the other coating properties. In order to understand these mechanisms and their interaction better, advanced XRD analyses should be carried out. It is also useful to record the velocity and temperature of the particles during flight in order to better understand the exact influence of the atomizing gas in the future.
